# Nutmeg Poisoning With Electrolyte Abnormalities: A Case Report

**DOI:** 10.7759/cureus.71840

**Published:** 2024-10-19

**Authors:** Ryo Ichibayashi, Takanobu Sato, Ryogo Ohashi, Katsuya Konishi

**Affiliations:** 1 Department of Internal Medicine, Division of Emergency Medicine, Sakura Medical Center, Toho University, Sakura, JPN; 2 Department of Dermatology, Sakura Medical Center, Toho University, Sakura, Sakura, JPN

**Keywords:** anticholinergic effects, electrolyte abnormalities, hyponatremia, nutmeg intoxication, poisoning

## Abstract

Nutmeg is a commonly used spice that contains myristicin, a compound with anticholinergic and psychoactive properties. Excessive consumption can lead to toxicity, causing psychiatric and anticholinergic symptoms such as tachycardia, dizziness, dry mouth, tremors, and nausea. Nutmeg poisoning is rare and not widely recognized. This report describes the case of a 33-year-old woman who accidentally ingested a toxic amount of nutmeg while cooking. She presented with symptoms of dry mouth, tremors, dizziness, and anxiety and was subsequently diagnosed with nutmeg poisoning. Blood tests revealed electrolyte abnormalities, while cardiac function and respiratory status were normal. The patient was treated with fluid restriction and symptomatic management, with symptoms resolving within 24 hours. This case highlights the need for greater awareness of nutmeg’s toxic effects and the importance of recognizing its symptoms and providing appropriate treatment. Nutmeg can pose significant health risks if consumed excessively despite its widespread availability. Education and prevention measures are crucial to avoid complications, including electrolyte abnormalities. Healthcare providers should consider nutmeg poisoning in differential diagnoses involving anticholinergic and sympathomimetic symptoms, as early recognition and management are essential to patient recovery.

## Introduction

Nutmeg is a spice used in household dishes to remove the odor of meat [1,2]. The myristicin in nutmeg has anticholinergic and psychoactive effects and may cause organ damage, such as liver, heart, and kidney damage [1,2]. As a result, excessive nutmeg intake can cause anticholinergic and psychiatric symptoms. Signs and symptoms include tachycardia, dizziness, excitement, anxiety, headache, limb weakness, dry mouth, and nausea [1,3].

There have been cases of people taking nutmeg in excess to achieve hallucinations and euphoria, but severe cases and deaths have also been reported due to taking large amounts [3,4]. There have also been reported cases of people taking excessive amounts of nutmeg in addition to smoking marijuana to achieve a euphoric effect [5]. In general, awareness of the poisoning symptoms of nutmeg is low [3]. In this report, we describe a case of nutmeg poisoning and electrolyte abnormalities caused by accidentally ingesting a large amount of nutmeg in a meal without being aware of its toxicity.

## Case presentation

A 33-year-old woman presented with symptoms nutmeg poisoning. She had no medical history or allergies. At around 6 p.m. earlier that day, she had eaten spaghetti with meat sauce; the inner cap of the nutmeg bottle came off during cooking, and two-thirds of the bottle's contents fell into the spaghetti. At around 9 p.m., she began to feel a dry mouth, and at the same time, she began to feel trembling and dizziness. She researched nutmeg online and found that the symptoms matched the nutmeg bottle's symptoms. She realized that she had consumed a toxic amount. As her mouth felt excessively dry, she panicked and drank more than 1 liter of water in 30 minutes. After that, she also experienced headaches, nausea, and discomfort in the epigastric region, and as the symptoms did not improve, she was brought to our hospital by ambulance.

On arrival, she was conscious, with a blood pressure of 131/91 mmHg, respirations of 24/minute, pulse of 105/minute, and saturation of peripheral oxygen (SpO2) of 100%. She complained of gagging and had a gross tremor in her left upper limb. There was no dry skin. The pupil diameter measured with a quantitative pupillometer was 5.8 mm on the right and 5.1 mm on the left, and a light reflex was observed. A chest X-ray and an electrocardiogram were normal. The total weight of the vial of nutmeg was 15 g, which means that the patient had ingested a toxic amount of 5-10 g. We diagnosed her with nutmeg poisoning because we believed her gagging, tachycardia, and dilated pupils were due to the anticholinergic effects of nutmeg. The blood test results at the time of admission are shown in Table 1.

**Table 1:** Laboratory results during her admission CRP: C-reactive protein, TP: total protein, Alb: albumin, AST: aspartate aminotransferase, ALT: alanine aminotransferase, LDH: lactate dehydrogenase, γ-GTP: γ-glutamyl transpeptidase, BUN: blood urea nitrogen,  eGFR: estimated glomerular filtration, WBC: white blood cell, Hb: hemoglobin, Ht: hematocrit, Plt: platelet, SG: specific gravity

Test	Day 1 Result	Day 2 Result	Day 3 Result	Reference range
CRP	0.02	-	-	<0.3 mg/dL
TP	7.3	-	-	6.7-8.3 g/dL
Alb	4.5	-	-	3.8-5.2 g/dL
AST	19	19	15	10-40 IU/L
ALT	13	11	11	5-45 IU/L
LDH	168	-	-	124-222 U/L
γ-GTP	33	46	47	<30 IU/L
BUN	12.9	7.8	12.1	8.0-20.0 mg/dL
Creatinine	0.59	0.52	0.74	0.47-0.79 mg/dL
eGFR	94	107	73	>60 mL/min/1.73m^2^
Sodium	128	128	138	137-147 mEq/L
Potassium	3.1	4.3	4.3	3.5-5.0 mEq/L
Chloride	96	98	107	98-108 mEq/L
Glucose	127	-	-	70-109 mg/dL
HbA1c	5.4	-	-	4.7～6.2％
WBC	7720	11060	4710	3300-9000/μL
Hb	12.7	13.0	13.8	13.5-17.5 g/dL
Ht	44.9	37.2	40.2	39.7-52.4%
Plt	36.5	20.3	19.4	14-3410^4^/μL
Urinalysis	-	-	-	-
pH	7.5	7.5	5.5	5.0～8.0
SG	1.013	1.003	1.011	1.010～1.025

Other than hyponatremia and hypokalemia, no other notable findings were noted. Cardiac ultrasound showed no abnormalities in cardiac function, and no respiratory changes were observed in the diameter of the inferior vena cava, so it was determined that there was no dehydration. However, the low urine specific gravity and the large amount of water consumed in a short period suggested water intoxication. After admission to the general ward, intravenous fluids were restricted to 500 mL/24 hours (Ringer's solution). The patient was monitored, and seven hours after ingesting nutmeg, tremors, restlessness, and anxiety disappeared. Twelve hours later, blood pressure improved to 103/66 mmHg and pulse rate to 78/min, normal. The dry mouth improved within 24 hours after ingestion, but epigastric discomfort persisted. On hospital day 2, electrolyte abnormalities improved, and symptoms resolved, so the patient was discharged. One week after discharge, mydriasis and electrolyte abnormalities were not noted. As a result, we have stopped following up on the patient.

## Discussion

Nutmeg poisoning can occur with ingestion of 5 g or more, leading to mental symptoms such as anxiety, hallucinations, and restlessness due to myristicin and other compounds in nutmeg [1,3,6,7]. Anticholinergic effects like dry mouth, tremors, tachycardia, and decreased vision may also appear, sometimes necessitating ambulance transport. Symptoms typically begin three hours post ingestion and subside within 48 hours [3,6]. These symptoms are not commonly found in general poisoning textbooks, prompting initial information gathering from the Internet. Despite abundant online details, awareness of nutmeg poisoning is low [3], emphasizing the importance of a patient’s medical history for accurate diagnosis.

In the reported case, the patient exhibited mydriasis with a light reflex, attributed to nutmeg's anticholinergic effects. Myristicin, contained in nutmeg, has anticholinergic effects [2]. Generally, anticholinergic poisoning results in mydriasis with loss of pupil reflex, while sympathomimetic drugs cause pupil dilation with pupil reflex. This contradiction indicates the insufficient elucidation of the pharmacological action of nutmeg [3]. The patient did not exhibit sweating or dry skin but complained of dry mouth. Rapid excess fluid intake was thought to be the cause of hyponatremia [8]. In this way, the anticholinergic dry mouth can lead to excessive water consumption, causing electrolyte abnormalities. In severe cases, it can also cause water intoxication. Metabolites of myristicin and elemicin in nutmeg are amphetamine derivatives, which have neurostimulant effects causing excitement and coma [9]. In the worst case, hyponatremia can worsen these neurological symptoms. Even if there is a dry mouth, excessive fluid therapy is unnecessary. Appropriate fluid therapy and symptomatic treatment are important.

Nutmeg poisoning presents a mix of anticholinergic and sympathomimetic effects, leading to sympathetic nervous system activation. There is no specific cure, but treatment involves body temperature control, rest, and mental health support unless the case is severe. Understanding nutmeg's effects and appropriate first aid for poisoning is vital. Patient education should discourage excessive water drinking and stress the importance of seeking medical attention to prevent complications like electrolyte abnormalities and water intoxication. Regarding pupillary findings, the presence or absence of light reflex may depend on which effect is more dominant. Differential diagnoses for symptoms like mydriasis, elevated blood pressure, tachycardia, and psychiatric symptoms include anticholinergic poisoning, sympathomimetic poisoning, and serotonin syndrome. Awareness of nutmeg poisoning’s dual effects can aid physicians in considering it as a differential diagnosis based on pupillary findings.

## Conclusions

Although nutmeg is readily available in homes, knowledge about its toxic risk and pharmacological effects must be improved. Diagnosis and treatment must rely on the medical history and clinical symptoms, and patients may present with electrolyte abnormalities. Prevention and education about nutmeg poisoning and its treatment are important to prevent unnecessary complications.
